# The impact of community and provider-driven social accountability interventions on contraceptive use: findings from a cohort study of new users in Ghana and Tanzania

**DOI:** 10.1186/s12939-023-01928-0

**Published:** 2023-08-28

**Authors:** Petrus S. Steyn, Joanna Paula Cordero, Dela Nai, Donat Shamba, Kamil Fuseini, Sigilbert Mrema, Ndema Habib, My Huong Nguyen, James Kiarie

**Affiliations:** 1 Development and Research Training in Human Reproduction, UNDP/UNFPA/UNICEF/WHO/World Bank Special Programme of Research, Avenue Appia 20, 1202 Geneva, Switzerland; 2 Population Council, 204 Yiyiwa Drive, Abelemkpe, Accra, Ghana; 3 Department of Health Systems, Impact Evaluation and Policy, Ifakara Health Institute, P.O.BOX 78373, Dar Es Salaam, Tanzania

**Keywords:** Social accountability, Contraception / family planning, Contraceptive use

## Abstract

**Background:**

Although contraceptive use has increased over 15 years, discontinuation rates remain high. Contraceptive use is becoming more important when addressing unmet need for family planning. Social accountability, defined here as collective processes for holding duty bearers to account for their actions, is a rights-based participatory process that supports service provision and person-centred care, as well as, informed decision-making among community members regarding their health. A study implemented in Ghana and Tanzania was designed to understand and evaluate how social accountability and participatory processes influences quality of care and client satisfaction and whether this results in increased contraceptive uptake and use. We report here on the relationship between social accountability and the use of modern contraceptives, i.e., contraceptive method discontinuation, contraceptive method switching, and contraceptive discontinuation.

**Methods:**

As part of Community and Provider driven Social Accountability Intervention (CaPSAI) Project, a cohort of women aged 15 to 49 years who were new users of contraception and accessing family planning and contraceptives services at the study facilities across both intervention and control groups were followed-up over a 12-month period to measure changes contraceptive use.

**Results:**

In this cohort study over a one-year duration, we did not find a statistically significant difference in Ghana and Tanzania in overall method discontinuation, switching, and contraceptive discontinuation after exposure to a social accountability intervention. In Ghana but not in Tanzania, when stratified by the type of facility (district level vs. health centre), there were significantly less method and contraceptive discontinuation in the district level facility and significantly more method and contraceptive discontinuation in the health centres in the intervention group. In Ghana, the most important reasons reported for stopping a method were fear of side-effects, health concerns and wanting to become pregnant in the control group and fear of side-effects wanting a more effective method and infrequent sex in the intervention group. In Tanzania, the most important reasons reported for stopping a method were fear of side-effects, wanting a more effective method, and method not available in the control group compared to wanting a more effective method, fear of side-effects and health concerns in the intervention group.

**Conclusions:**

We did not demonstrate a statistically significant impact of a six-month CaPSAI intervention on contraceptives use among new users in Tanzania and Ghana. However, since social accountability have important impacts beyond contraceptive use it is important consider results of the intermediate outcomes, cases of change, and process evaluation to fully understand the impact of this intervention.

**Trial registration:**

The CaPSAI Project has been registered at Australian New Zealand Clinical Trials Registry (ACTRN12619000378123, 11/03/2019).

## Background

In Sub-Saharan Africa, modern contraceptive uptake and use are rising, albeit with considerable geographic variation [1]. The reasons for the rise in modern contraceptive prevalence rates include increased access to and use of long-acting reversible contraceptive options, postpartum contraceptive methods, and community health workers [1]. Using data from Demographic and Health Survey (DHS) reports, the Performance Monitoring and Accountability 2020 project, as well as the United Nations world contraceptive database on contraceptive use, it was reported that although contraceptive use increased over 15 years, discontinuation rates were high [1].

### Family planning and importance of method continuation, method switching, and contraception continuation

Contraceptive use is becoming more important when addressing unmet need for family planning [2]. Contraceptive use encompasses method continuation,—discontinuation,—switching, and contraceptive discontinuation. Several factors may influence contraceptive use, such as interpersonal communication and patient-provider communication. The importance of switching to another method has been illustrated by Jain et al. [3]. In a study where they collected data on 36 low and middle-income countries (LMIC) on the use of contraceptives by married women, it was estimated that 38% of unmet need for contraception was due to discontinuation of contraceptive methods among those who desired to avoid pregnancy [4]. This means that contraceptive discontinuation contributes to a huge number of unintended pregnancies. Contraceptive discontinuation accounts for one-third of unintended pregnancies [4]. A way to reduce this unmet need is to reduce contraceptive discontinuation by enabling switching to another effective contraception option.

Contraceptive method discontinuation is not inevitable, based on the reasons given in 45% of the incidents [3]. These include side-effects, wanting a more effective method, lack of access, high costs, inconvenience, infrequent sex/husband away, difficulty getting pregnant/menopausal, marital dissolution/separation [3]. This was confirmed by Ali M et al. [5] in a study on causes and consequences of contraceptive discontinuation using 60 Demographic and Health Surveys. The authors reported that many women who use reversible modern methods do so inconsistently or discontinue use because they are not satisfied with the method, are concerned about the side effects, or have trouble getting supplies [5]. Sully et al. [6] reported dissatisfaction, side-effects, and supply problems as reasons for inconsistent use or discontinuation of the method among women who use reversible modern methods. The duration of method effectiveness also affects continuation as was shown by data for 33 LMICs where 20% of users of short-acting methods who want to avoid pregnancy discontinue use within the first year against 11% of women who used intrauterine device and 8% of women who used contraceptive implants [7].

Strengthening the quality of contraceptive information, education, and counseling services seems implicit, as is providing long-acting contraception to enhance use-effectiveness [1]. High-quality services, including counseling, to women using methods, are as important as adding new users towards meeting unmet need (3*).* Person-centered care (PCC) is where provider-person (client) relationships, effective communication, and shared-decision making are advocated. High-quality PCC improves both women's experience and may also lead to better outcomes [8]. Some measures of PCC quality appear to impact contraceptive continuation. A systematic review reported mixed findings on relationships between PCC and clinical outcomes, with stronger evidence for positive influences of PCC on satisfaction and self-management, but a lack of understanding of how specific PCC processes relate to patient outcomes [9].

A recent systematic review of interventions focused on PCC for family planning found that most interventions were successful in increasing client knowledge about family planning and overall experience, but results were mixed for family planning uptake and continuation [10]. Furthermore, other studies reported that perceived quality of family planning care is associated with client satisfaction and method and contraceptive continuation [11, 12].

### The role of social accountability to change interpersonal care and health behaviours

Social accountability, defined here as "citizen-led collective processes for holding duty bearers, including politicians, government officials, and/or service providers, to account for their actions" [13], is a rights-based participatory process that supports service provision and PCC, as well as, informed decision-making among community members regarding their health. Thus, social accountability processes could potentially ensure that family planning services are responsive to client needs and promote trust between women and girls and the health system [14]. Several studies in reproductive, maternal, newborn, child and adolescent health (RMNCAH) demonstrated positive results, especially in intermediate outcomes such as enhanced infrastructure service delivery, commodities and resources allocation, and service utilization [15–18]. Several studies have reported positive outcomes of social accountability for contraceptive programs such as improvements in service quality, financial allocation for service provision [16, 19], community awareness and participation [20–24] and increased use of modern methods [23]. Social accountability is therefore well suited to support an enabling environment for family planning programs.

The Community and Provider driven Social Accountability (CaPSAI) Project was designed to understand and evaluate the effects of social accountability and participatory processes in the context of a family planning and contraceptive (FP/C) program. The study design, aims, and theory of change are described in detail a protocol manuscript and the Australian New Zealand clinical trial registry [24, 25]. To summarise, this was a complex-designed study exploring how a social accountability process in the context of FP/C programs/services influences QoC and client satisfaction and whether this results in increased contraceptive uptake and use. The design followed the Medical Research Council (MRC) guidance on measuring complex interventions and was grounded on a theory of change and a co-designed intervention. [24, 26, 27]. It accounts for the multiple components to track the levels and interrelated outcomes and includes a process evaluation component [24].

**Fig. 1:**
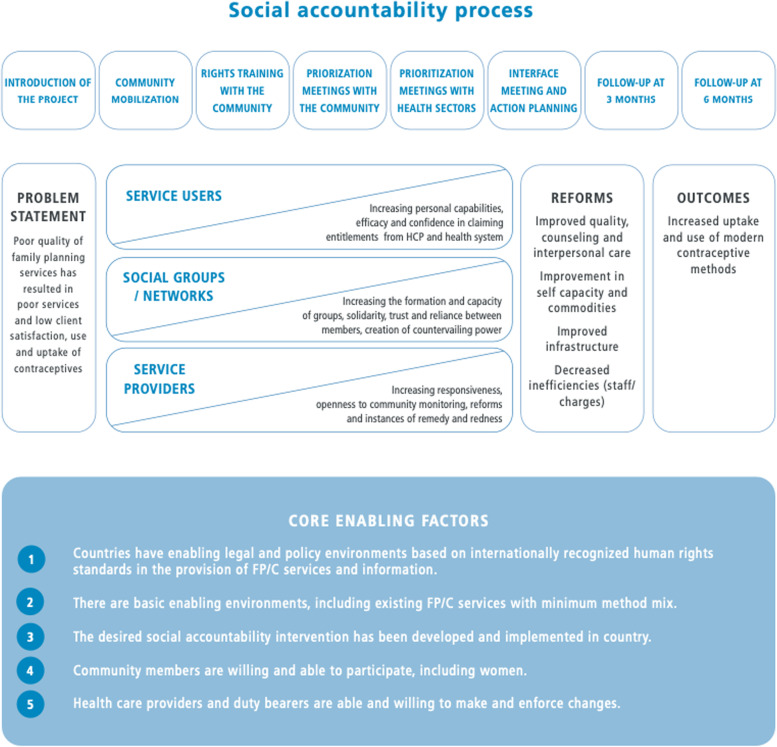
CaPSAI Theory of Change [29]

The development of the theory of change (ToC) has been described thoroughly elsewhere [18, 24] (Fig. 1). Briefly, development followed a literature review [28] and findings from a formative phase [17, 24]. Eight core steps were identified as the base of the intervention, which involved community members and health system actors identifying challenges in service provision and care, developing plans of action to improve quality of services, counseling, interpersonal care, staff capacity, and commodity availability [24]. According to ToC, these would lead to positive changes in contraceptive use, such as less contraceptive discontinuation and higher rates of method switching if discontinued.

This manuscript reports on the findings of the CaPSAI cohort study, which aimed to measure changes in behaviors around contraceptives.

## Methodology

### Study design

As part of CaPSAI, a cohort of women aged 15 to 49 years who were new users of contraception and accessing family planning and contraceptives services at the study facilities across both intervention and control groups were followed-up over a 12-month period to measure changes contraceptive use [24]. The outcomes measured include: contraceptive method continuation defined as the proportion of women in the cohort using the same contraceptive method after 12 months; contraceptive switching, the proportion of women initiating a method that change methods within 12 months; and contraceptive discontinuation, the proportion of women initiating contraception who stopped using any method within 12 months.

### Study setting

The CaPSAI Project was implemented in Ghana and Tanzania. In Ghana, modern contraceptive prevalence rate (mCPR) among currently married women increased from 18.7% in 2003 to 22.2% in 2014 [30] and 25.0% in 2017 [30]. Further, the contraceptive discontinuation rate was 25% within 12 months of starting its use for any reason [31]. In Tanzania mCPR increased steadily from 23.0% in 2004/2005, 30.8% in 2010 and 34.3% in 2015/2016 [32, 33]. The overall use of modern contraceptives increased by 11.3 percent point. A higher increase in modern contraceptive use was detected between 2004—2010, a 7.8%-point increase compared with a 3.5%-point increase detected between 2010 – 2016 (3130). See trend in use of modern contraceptive methods among currently married women in Ghana and Tanzania (Table 1) In Tanzania, 26% of women using a contraceptive method discontinued the method in less than 12 months [33]. In 6% of episodes, women switched to another method.

**Table 1:** Trends in current use of modern contraceptive methods among currently married women and discontinuation rates of modern contraceptives in Ghana and Tanzania

Ghana				Tanzania		
**Use of modern Contraception**	19.5%			32.0%		
**Contraceptive discontinuation rate**	25%			26%		
**Switching between methods**	Not available			6%		
Trend in current use of modern contraceptive methods among currently married						
**Ghana**						
**Years**	**1988**	**1993**	**1998**	**2003**	**2008**	**2014**
mCPR	5.2	10.1	13.3	18.7	16.6	22.2
**Tanzania**						
**Years**				**2004/2005**	**2010**	**2015/2016**
mCPR				20.0%	27.0%	32.0%

In Ghana, the study was conducted in the Central region. Four districts, namely Gomoa East, Agona West, Ekumfi, and Ajumako, were selected for the control group. The intervention group had three districts, which were Abura-Asebu-Kwamankese, Komenda-Edna-Eguafo Abirem, and Mfatsiman. In Tanzania, two districts, Mbeya and Chunya, were selected for the intervention group. Two districts, Iringa Urban and Iringa Rural, were selected to be the control group. In Ghana the health centre/clinic and Community based health planning and services were at the primary level health care and the district hospitals were at the second level of health care. In Tanzania the dispensary and health centre were at the primary level health care and district hospital and regional referral hospitals were at the secondary health care. A summary of the demographic information on the facilities chosen is provided in Table 2.

**Table 2:** Demographic information of the facilities chosen Ghana and Tanzania

Facility nr	Group 1 = Intervention 2 = Control	Level of facility	District Name	Type of location (Urban/Semi-urban/Rural)	Average no. of clients (women of reproductive age 18–49), per month from the 3 months estimates	Average no. of new users of modern contraception, per month ( (ESTIMATED from the 6 months figures)	Number of women aged 15–49 in the facility catchment area
**Ghana**							
1	1	Primary	Abura-Asebu-Kwamankese	Urban	93	80	3225
2	1	Primary	Abura-Asebu-Kwamankese	Rural	59	58	739
3	1	Primary	Abura-Asebu-Kwamankese	Urban	253	139	5016
4	1	Primary	Komenda-Edna-Eguafo-Abirem	Urban	65	52	7569
5	1	Secondary	Komenda-Edna-Eguafo-Abirem	Urban	170	90	4073
6	1	Primary	Komenda-Edna-Eguafo-Abirem	Urban	120	55	10,893
7	1	Primary	Komenda-Edna-Eguafo-Abirem	Urban	65	52	7569
8	1	Primary	Mfantsiman	Urban	74	55	7752
9	2	Primary	Agona West	Urban	121	56	5314
10	2	Secondary	Agona West	Urban	216	76	15,428
11	2	Primary	Agona West	Urban	295	101	778
12	2	Primary	Gomoa East	Urban	167	47	2708
13	2	Primary	Gomoa East	Urban	220	142	15,343
14	2	Primary	Gomoa East	Rural	98	87	5807
15	2	Primary	Ekumfi	Urban	92	61	5691
16	2	Primary	Ajumako-Enyan-Essiam	Urban	96	58	8385
**Tanzania**							
1	2	Urban/Primary	Mbeya City Council	Urban	124	91	1392
2	2	Urban/Primary	Mbeya City Council	Urban	177	218	2164
3	2	Urban/Primary	Mbeya City Council	Urban	211	130	1425
4	2	Urban/Primary	Mbeya City Council	Urban	563	654	6078
5	2	Urban/primary	Mbeya City Council	Urban	358	347	2044
6	2	Rural/Primary	Chunya District Council	Rural	426	310	2780
7	2	Rural/Primary	Chunya District Council	Rural	151	238	5661
8	2	Rural/Primary	Chunya District Council	Rural	387	293	6573
9	1	Rural/Primary	Iringa District council	Rural	211	63	1029
10	1	Rural/primary	Iringa District council	Rural	606	434	1297
11	1	Rural/Primary	Iringa District council	Rural	380	263	5273
12	1	Urban/Primary	Iringa Municipal Council	Urban	442	426	10,169
13	1	Urban/Primary	Iringa Municipal Council	Urban	938	505	15,904
14	1	Urban /Primary	Iringa Municipal Council	Urban	260	132	6422
15	2	Urban/Primary	Iringa Municipal Council	Urban	198	306	2910
16	2	Urban/Primary	Iringa Municipal Council	Urban	95	134	1702

The implementing partners had not previously implemented their social accountability programmes in the selected districts in the two countries. The selection of districts was also based on similarities in cultural, religious, and socio-economic factors. Following a mapping of facilities in the districts, eight facilities in intervention districts and eight facilities in control districts were selected in each country. Criteria for selecting facilities included type and level, the average number of service users, number of new users, and matching by these criteria between facilities in intervention and control districts.

### The intervention

CaPSAI used a co-design process to define the intervention of study [34]. This means that the intervention processes in the two study countries are not identical, but all contain the standard steps identified in the theory of change (Table 3). The standard steps are described in more detail elsewhere [18, 24, 34]. Table 1 summarizes the activities conducted for each of the steps in each country. The implementation of the intervention started end of April or beginning of May 2018 with Step 1. Step 8 was completed in December 2018.

**Table 3:** Intervention activities in each study country

Step	Ghana	Tanzania
1. Introduction of the intervention to the community	• Consultative meetings with key regional and district health stakeholders to secure buy-in • Consulted the District Social Welfare Department to ensure inclusion • Consultative meetings with facility and community stakeholders, e.g., Chiefs, Assemblymen • Identified and met with local partners, including local non-governmental organizations, community-based organizations, or self-help groups, to get buy-in	• Introduction meeting with local leaders (Ward Development committee (WADC) members, influential/religious leaders, Health Facility Governing Committee (HFGCs) and village leaders, and health facility meetings
2. Mobilization of participants for the intervention	• Facilitators and community partners with implementation staff convened (announcement, key speakers, prepare slides, etc.), held eight community meetings (including health providers) across the three districts to introduce the project, its approach, and arranged for rights and civic education activities • Selected five members for a Project Advisory Committee (PAC) at the district (facility/community) level for each district • Facilitators and community partners mobilized the community for the next step	• Meeting with women's groups, youth's groups, people with disabilities, pastoralist communities, community-based organizations, etc • Community meetings—recruitment of Social Accountability Monitoring (SAM) Team members (maximum of 20 SAM team members based on the criteria developed) • Health facility meetings – participant recruitment • Peer-to-peer mobilization of local organizations, women's groups • Distribution of fliers and other promotional materials to target groups
3. Health, rights, and civic education with community participants	• Facilitators and community partners convened the meeting, and family planning expert and GII staff conducted eight trainings across the three districts on the family planning standards, good governance and accountability in Ghana (Clients and service providers). Lasted four to six hours and was conducted on the same day as community card scoring	• A 3-day training for the participants (SAM cycle, health rights and civic education) • Field verification – health facilities/Community visit
4. Prioritization meeting with community	• Facilitators and community partners held prioritization workshop per district on family planning standards, good governance for service users and discussed existing services as against right-based standards • Separated participants into smaller groups (e.g. young women, men etc.) to identify issues • Collectively consolidated indicators for prioritized issues • Collectively ranked issues and agreed on the score • Talked about the next steps, including nominating people to speak at interface meeting	• Group discussions in each ward/health facility attended by SAM Team and community members to prioritize issues • Compilation and merging of priorities from discussion into one document (by the representative from each group) • Feedback on consolidated priorities to the bigger group (Health facility/ward)
5. Prioritization meeting with duty bearers	• Implementation staff, a family planning expert, and facilitators held dialogue meetings in all districts on standards, good governance with service providers to discuss existing services as against rights-based standards • Identified issues (e.g., in a group or individual basis or some combination) • Agreed on indicators for prioritized issues • Ranked the issues and agreed on the scores • Talked about the next steps, including arrangements for pre-interface meeting	• Training of the health workers on family planning standards and Rights • Group meeting with health facility and facilitators to assess the provision of FP services at the health facilities • Discussion and scoring of the priorities/issues • Compilation and merging priorities to be shared in the interface meetings
6. Interface meeting and joint action planning	• Coordinated the eight interface meetings with providers and users and other relevant officials shortly after the prioritization meeting facilitated by implementation team staff, facilitators, and community partners • Groups developed joint action plans for the next three months and six months • Agreed that community members will monitor implementation	• Presentation of the priority lists from the two groups • Discussion and scoring of the combined list • Development of an action plan (jointly) •Share Action Plan with community participants, Service Providers, local government authorities (LGA) leaders, and Executives • Interface meetings conducted in each district
7. First follow-up meeting with community and duty bearers at three months	• Convened follow-up interface meeting with service providers and users to assess changes in the scores and follow up progress on the action plan and make any changes • Regular monthly phone calls conducted with community monitors • A tool for capturing data to track changes occurring in behaviour at facility with service providers and service users was developed and used by community monitors	• Monitor the progress of the agreed Action Point monthly • SAM Task force team conducting community feedback meeting on the progress • Share the progress of the Action Plan with the LGA executives
8. Second follow-up meeting with community duty bearers at six months	• Convened follow-up interface meeting with service providers and users to assess changes in the scores and follow up progress on the action plan and make any changes • Continued monitoring using tool by community monitors and follow-ups with duty bearers at district level	• Monitor the progress of the agreed Action Point monthly • SAM Task force team conducting community feedback meeting on the progress • Share the progress of the Action Plan with the LGA executives

### Sample size

The discontinuation rate, a time-to-event outcome with censoring, required the use of sample size estimation methods for survival data. As described in the protocol [24, 25], the sample size estimation was computed using values of hazard ratio (intervention vs. control) of 0.5, 0.6, and 0.7, and given values of the proportion of discontinuing use of modern contraception by the end of the first year, in the control group ranged from 30 to 60%. The estimates were obtained using a Type I error at 5% level, accrual time of 0.01 years, and an exponential loss to follow-up of 20% that enables a two-sided Logrank test to achieve 80% statistical power to detect the difference in discontinuation rates by the end of one year of follow-up. Adjustments for clustering were made on the final sample size since the intervention was not at the individual level. Using an intra-class correlation of 0.05 and an average cluster size of 20 first users of modern contraception resulting in a design effect of 2.0, the final sample size was doubled. The sample was estimated to be 800 women across five to eight study facilities per group. The total sample was 1600 women distributed according to the size of the facilities in the intervention and control groups in each country.

### Data collection

Data collection was done in real-time using tablet-based standardized interview questions developed using OpenClinica Participate. During data capture, the electronic data capture system performs edit checks to immediately notify of potential errors and inconsistencies. The local study team kept an updated log of screened and enrolled study participants per the data management standard operating procedure [35].

The two main instruments (intake and follow-up interview) were used to collect data from new users of family planning services at the facilities [36]. A mid-term check-up interview at six months following the intake interview was conducted to minimize loss to follow-up and to confirm if the participants are still using a method and which method it is to estimate continuation [36]. The cohort study instruments were adapted from existing tools. The Demographic Health Survey model questionnaires were used to capture demographic characteristics and contraceptive use [29]. Questions from the MEASURE Evaluation's Quick Investigation of Quality were used to capture outcomes related to client satisfaction [37]. The follow-up questionnaire used by Barden-O'Fallon et al. in a one-year study on contraceptive continuation was adapted [38]. The subject areas covered included an update on socio-demographic characteristics, contraceptive use or pregnancy status or intention, experience with side effects, and reproductive and household decision-making [38]. Exposure to the intervention or knowledge among study participants was also captured.

### Participant selection, enrolment, and follow-up

Possible study participants were identified during consultations by health providers who referred them to study staff. Screening was done by the study staff using the eligibility criteria that included age [15–19] and whether they were initiating a modern contraceptive method for the first time, switching from a traditional method, or restarting a method after six months of not using one. Following the consent or assent process, the intake interviews were conducted in person at the facility, or an appointment was set later. For the check-up, study staff contacted study participants who did not come to the facility by telephone to conduct the interview or invite them to come to the facility. For study participants who could not be contacted by phone during the check-up at six months, a letter was delivered inviting them to contact the study team. Appointments are set for the 12-month follow-up during the enrolment. If the study participants missed their appointments, they were contacted by phone to set a new appointment.

The intake interviews were conducted after completion of the main steps of the intervention, namely following the completion of the interface meeting (Step 6), where community members and duty bearers get together to jointly develop action plans based on prioritized issues. In both study settings, data collection for the intake interview started in October 2018 and was completed in December 2018. The follow-up interviews were conducted 12 months later, from October to December 2019. The interim check-up interview was done from May to July 2019.

To minimize loss to follow-up, experienced data collectors were recruited from the study areas who were available for the entire study period. Participants who missed their interview appointments were contacted by phone to remind them of the appointment or a study team member went to their homes if they consented to it. If participants could not be reached by phone, a letter was sent to the participant via a community health nurse, health worker or data collector in accordance with the approved protocol. Additionally, the project supported participants’ travel costs to the facility after each interview.

### Statistical methods

The rate of loss to follow-up for the one-year follow-up period was computed [24]. The one-year cumulative method discontinuation and method switching rates were compared between the intervention and control groups. Because of the clustered nature of the outcomes, with the intervention package designed at the cluster level, all time-to-event outcomes, including loss to follow-up, method discontinuation, and method switching, were analyzed with hazard ratios estimated from the shared frailty models. The multivariable frailty model was applied to adjust for potential baseline confounders at the participant and/or facility level. Unadjusted and adjusted hazard rate ratios (HRRs) were reported. Interaction between factors was assessed. Two-sided tests, 5% significance levels, and 95% confidence intervals were used for all relevant parameters. Statistical Analysis System (SAS) Version 9.4 and R Version 3.3.3 software packages were used for the statistical analyses.

## Results

### Follow-up status

**Fig. 2:**
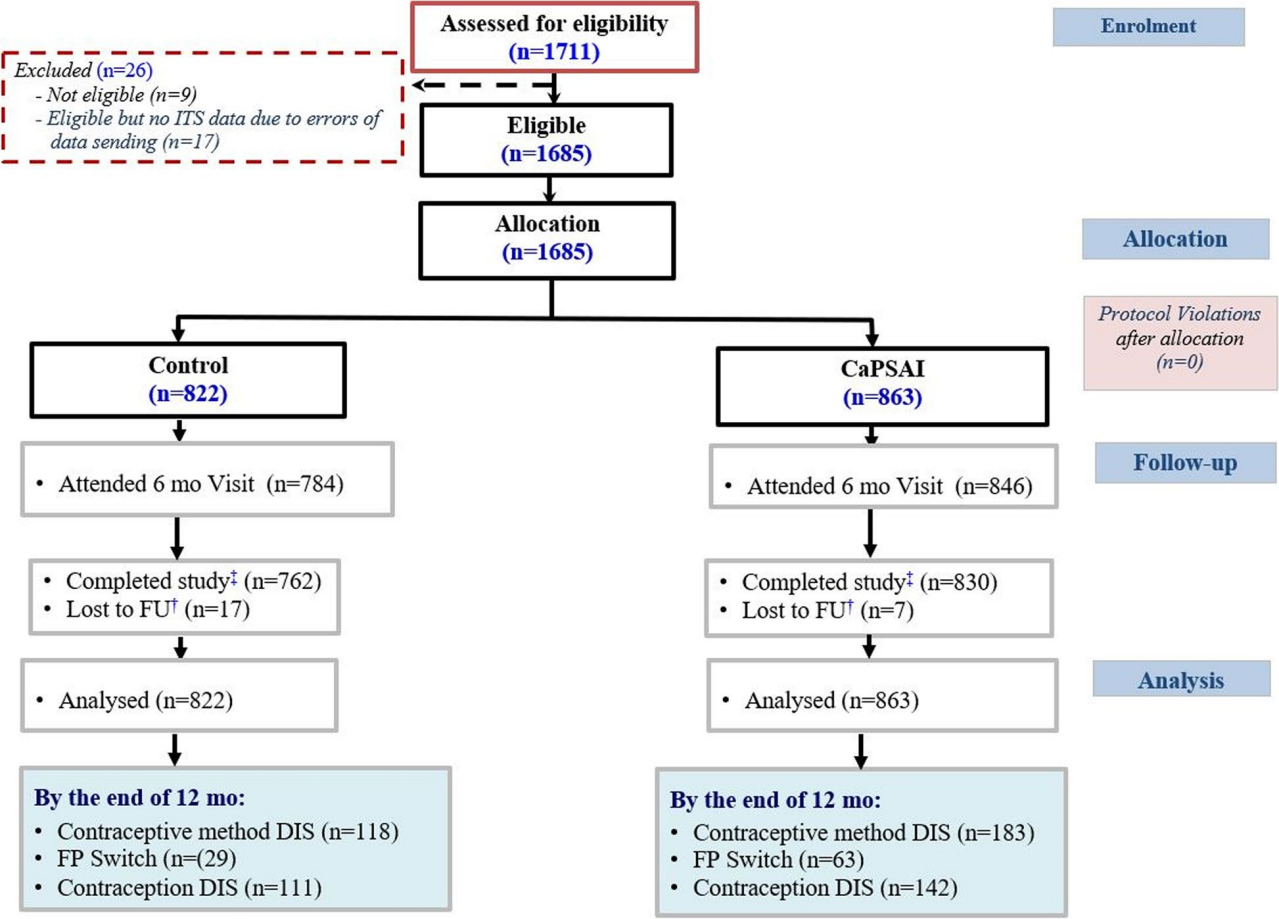
Ghana enrollment flowchart

In Ghana, 1,711 women were screened for eligibility (Fig. 2, 1,685 were eligible, 822 in the control and 863 in the intervention group. In the control group, 762 completed the study with 17 lost to follow-up. In the intervention group, 830 completed the study, and seven were lost to follow-up.

**Fig. 3:**
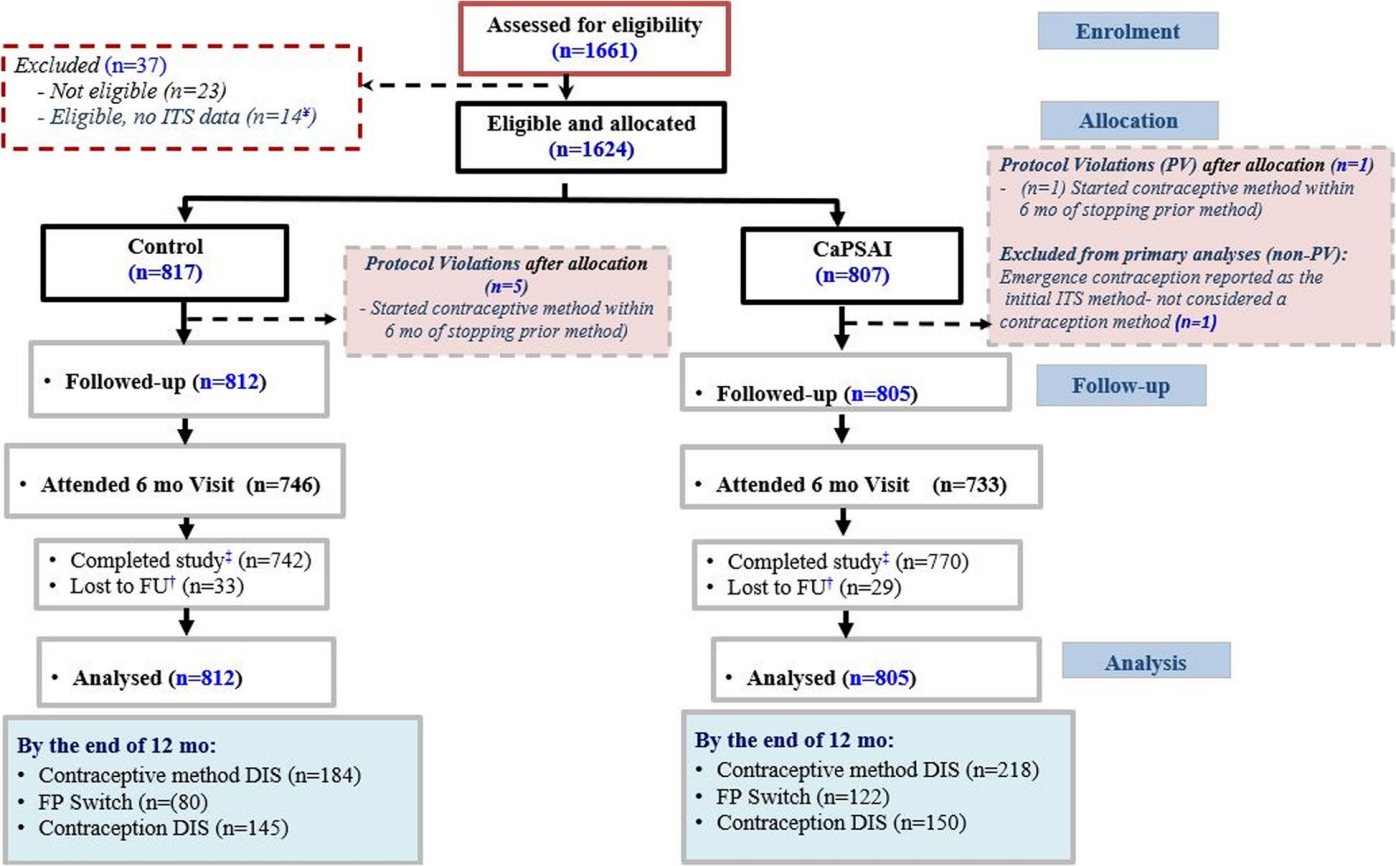
Tanzania enrollment flowchart

In Tanzania, 1,661 women were screened, and 1,624 were eligible (Fig. 3). Of 817 women enrolled in the control group, 742 completed the study, 28 were lost to follow-up, and five were excluded due to a protocol violation (enrolled less than six months of stopping a previous method). In the intervention group, of 807 women enrolled, 770 completed the study, 27 were lost to follow-up, and two were excluded due to protocol violation (one reported initiating a method less than six months of stopping a previous method and another initiated emergency contraception).

### Demographic characteristics

The demographic characteristics of the study population were comparable among intervention and control groups in both countries, as summarized in Table 2. In Ghana, the majority of women were 21 to 35 years of age, 75.6% and 67.2% in the control and intervention groups, respectively. In Tanzania, the 21 to 35 age group also made up the majority of the study population, 76.5% and 71% in the control and intervention groups, respectively. Majority of participants in both countries were married and not significantly different between the intervention and control groups, 54.3% in the control and 53.3% in the intervention in Ghana, 79.3% in the control and 80.3% in the intervention group in Tanzania. The distribution of the number of living children among women in the control and intervention groups was also similar in both countries.

### Method initiated

A quota for the type of method was not imposed during enrolment. In Ghana, the most popular method initiated was the injectable in both control (62.7%) and intervention group (70.9%) followed by implants, by 30.3% and 23.5% of the respondents in the control and intervention groups, respectively. Contraceptive pills were the third most commonly initiated method in both groups (3.3% in the control and 3.5% in the intervention). There were significantly more injectables initiated in the intervention group and more implants in the control group. In Tanzania, the most popular method initiated during the intake interview was the Implant in both control (58.1%) and intervention group (52.2%), followed by injectables used by 28% and 27% of the respondents in the control and intervention groups, respectively. Contraceptive pills were the third most commonly initiated method in both groups (7.1% in the control and 14.9% in the intervention). There were no statistically significant differences between the study and control group in methods initiated.

Several questions to understand informed choice were asked during the intake interview. In Ghana (Table 4), significantly more participants in the intervention group reported that they were told by a health or family planning provider about other methods of family planning that they could use (71.9% vs. 42.9%; (*p* ˂ 000.1) and significantly more participants in the control group reported that the provider described possible side effects of the specific method they chose (90.3% vs. 84.1%; *p* = 0.0002). There were no significant differences in the number of women who reported that the healthcare provider gave them a chance to ask questions and that the healthcare provider responded to the questions that they asked in the control and intervention groups.

**Table 4:** Ghana socio-demographic characteristics

Variables	Control *n* = 822 (%)	Intervention *n* = 863 (%)
**Age (intake interview)**		
15–20	127 (15.5)	177 (20.5)
21–35	621 (75.6)	580 (67.2)
36 + over	74 (9)	106 (12.3)
**Marital status (intake interview)**		
Currently married	446 (54.3)	460 (53.3)
Never married	343 (41.7)	375 (43.4)
Widowed, Separated or Divorced	33 (4.0)	28 (3.3)
**Number of living children (among those with live births) (intake interview)**		
0	120 (14.6)	162 (18.8)
1	213 (25.9)	220 (25.5)
2	224 (27.3)	161 (18.7)
3	124 (15.1)	141 (16.3)
4 +	141 (20.1)	179 (20.7)
**Method initiated (intake interview)**		
IUD	30 (3.65)	15 (1.74)
Injectables	515 (62.65)	612 (70.92)
Implants	249 (30.29)	203 (23.52)
Pill	27 (3.28)	30 (3.48)

In Tanzania (Table 5), there were significantly more participants in the control group who reported that: they were told by a health or family planning provider about other methods of family planning that they could use (84.5% vs. 67.1%; *p* ˂ 000.1), the provider described possible side effects of the specific method they chose (85.6% vs. 73.0%; *p* ˂ 000.1), the provider informed them what to do if they had any problems for the method they just accepted (93.0% vs. 78.4%; *p* ˂ 000.1), the healthcare provider gave them a chance to ask questions (76.8% vs. 53.6%; *p* ˂ 000.1) and the healthcare provider responded to the questions that they asked (97.6% vs. 92.8%;*p* ˂ 000.1).

**Table 5:** Tanzania socio-demographic characteristics

Variables	Control *n* = 812 (%)	Intervention *n* = 805 (%)
**Age (intake interview)**		
15–20	118 (14.5)	173 (21.4)
21–35	621 (76.5)	571 (71)
36 + over	73 (9)	61 (7.6)
**Marita status (intake interview)**		
Currently married	644 (79.3)	646 (80.3)
Never married	144 (17.7)	125 (15.5)
Widowed, Separated or Divorced	24 (3.0)	34 (4.2)
**Number of living children (among those with live births) (intake interview)**		
0	13 (1.6)	10 (1.2)
1	287 (35.5)	286 (35.5)
2	227 (28.0)	261 (32.4)
3	165 (20.3)	117 (14.5)
4 +	120 (14.8)	131 (16.3)
**Method initiated (intake interview)**		
IUD	34 (4.2)	17 (2.1)
Injectables	235 (28.9)	217 (27.0)
Implants	472 (58.1)	420 (52.2)
Pill	58 (7.1)	120 (14.9)

### Knowledge and exposure to the intervention

Knowledge of and exposure to the intervention remained low in the intervention groups of both Ghana and Tanzania (Table 6 and Table 7). In Ghana, of 118 women who knew of community monitoring and social accountability interventions, 112 had heard of CaPSAI activities, and 56 of 57 who participated in any type of community monitoring and social accountability activities participated in CaPSAI activities. In Tanzania, of 25 who knew about community monitoring and social accountability activities, eleven knew of CaPSAI activities. Only Three women reported participating in CaPSAI activities during the intake interviews in Tanzania.

**Table 6:** Informed choice and knowledge or exposure to social accountability interventions in Ghana

Informed choice	Control *n* = yes (%) (*n* = total responses)		Intervention *n* = yes (%) (*n* = total responses)	
Were you ever told by a health or family planning worker about other methods of family planning that you could use?	353 (42.9%) (*n* = 822)		*621 (71.96%) (*n* = 863) *(*p* ˂ 000.1)	
For the method you just decided to accept, did the provider describe possible side effects?	*708 (90.3%) (*n* = 784)		719 (84.1%) (*n* = 855) *(*p* = 0.0002)	
For the method you just decided to accept, did the provider tell you what to do if you have any problems?	760 (93.8%) (*n* = 810)		767 (90.1%) (*n* = 851)	
Did the healthcare provider give you a chance to ask questions?	673 (87.1%) (*n* = 773)		701 (84.4%) (*n* = 831)	
Did the healthcare provider respond to any questions that you asked?	661 (98.2%) (*n* = 673)		674 (96.2%) (*n* = 701)	
Was there anything from your consultation that you didn’t understand? [No]	767 (93.3%) (*n* = 822)		818 (94.8%) (*n* = 863)	
**Knowledge or exposure to social accountability interventions**	**Intake interview**	**Follow-up at 12 months**	**Intake interview**	**Follow-up at 12 months**
Knowledge of community monitoring and social accountability activities	14	8	118	163
Knowledge of CaPSAI intervention	0/14	0/8	112/118	151/163
Participation in community monitoring and social accountability activities	3	2	57	86
Participation in CaPSAI intervention	0/3	0/2	56/57	83/86

* Statistically significant <0.05

**Table 7:** Informed choice and knowledge or exposure to social accountability interventions in Tanzania

Informed choice	Control *n* = yes (%) (*n* = total responses)		Intervention *n* = yes (%) (*n* = total responses)	
Were you ever told by a health or family planning worker about other methods of family planning that you could use?	*690 (84.5%) (*n* = 817)		541 (67.1%) (*n* = 807) *(*p* ˂ 000.1)	
For the method you just decided to accept, did the provider describe possible side effects?	*697 (85.6%) (*n* = 814)		588 (73.0%) (*n* = 805) *(*p* ˂ 000.1)	
For the method you just decided to accept, did the provider tell you what to do if you have any problems?	*760 (93.0%) (*n* = 817)		631 (78.4%) (*n* = 805) *(*p* ˂ 000.1)	
Did the healthcare provider give you a chance to ask questions?	* 627 (76.8%) (*n* = 627)		431 (53.6%) (*n* = 804) *(*p* ˂ 000.1)	
Did the healthcare provider respond to any questions that you asked?	*612 (97.6%) (*n* = 673)		400 (92.81%) (*n* = 431) *p = 0.0002	
Was there anything from your consultation that you didn’t understand? [No]	749 (91.7%) (*n* = 822)		748 (92.7%) (*n* = 807)	
**Knowledge or exposure to social accountability interventions**	**Intake interview**	**Follow-up at 12 months**	**Intake interview**	**Follow-up at 12 months**
Knowledge of community monitoring and social accountability activities	23	3	25	19
Knowledge of CaPSAI intervention	0	0/3	11/25	8/19
Participation in community monitoring and social accountability activities	3	2	3	8
Participation in CaPSAI intervention	0/3	0/2	3/3	5/8

^*^ Statistically significant < 0.05

### Use of same facility at intake as compared to follow-up interview

In Ghana, 508 (61.8%) participants in the control and 584 (67.7%) participants in the intervention group attended the same clinic throughout the follow-up period. In Tanzania, 245 (30.2%) participants in the control and 324 (40.2%) participants in the intervention group attended the same clinic throughout the follow-up period.

In Tanzania, 15.01% in the control group and 21.5% in the intervention group were not attending the same facility during the follow-up compared to the intake interview. The main reason for not attending the same facility is that they moved out of area (50.89% in the control vs 50.60% in the intervention). They found the facility difficult to reach (30.36% in the control vs 33.13% in the intervention). Meanwhile, in Ghana, 5.91% and 11.93% were no longer attending the same facility as the intake in the control and intervention groups, respectively. In Ghana, the reasons for changing facilities were moving out of the area (44.44% in the control vs 22.22% in the intervention), not being able to make appointment" (2.22% in the control vs 34.34% in the intervention), and difficulty of reaching the facility (20% control vs 4.04% in the intervention).

### Contraceptive method discontinuation

**Fig. 4:**
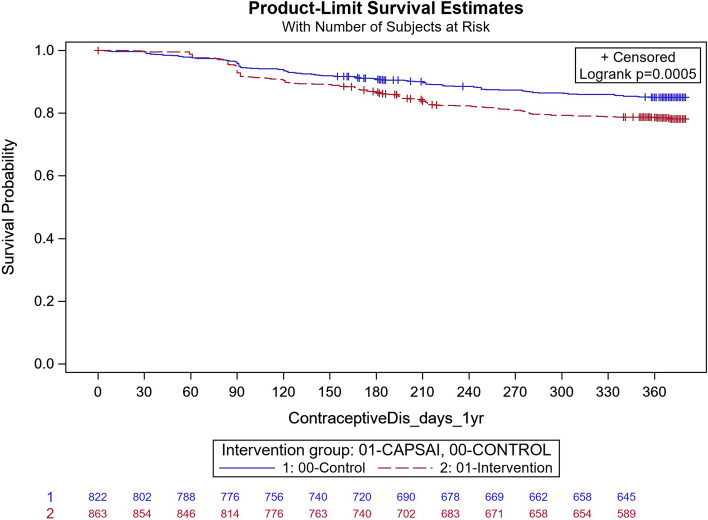
Ghana: overall contraceptive method discontinuation by 12 months. CaPSAI group: 183 events / 863 at risk and KM survival probability 78.1 (75.8,81.4). Control: 118 events / 822 at risk and KM survival probability 85.1 (82.4,87.4). Frailty model unadjusted HRR CaPSAI vs Control 1.18 (0.59, 2.37), *p*-value = 0.64

The contraceptive method discontinuation is the proportion of women not using the method that they started after one year of initiation and is expressed in Fig. 4 to Fig. 7 as time-survival estimates of the intervention against the control group.

**Fig. 5:**
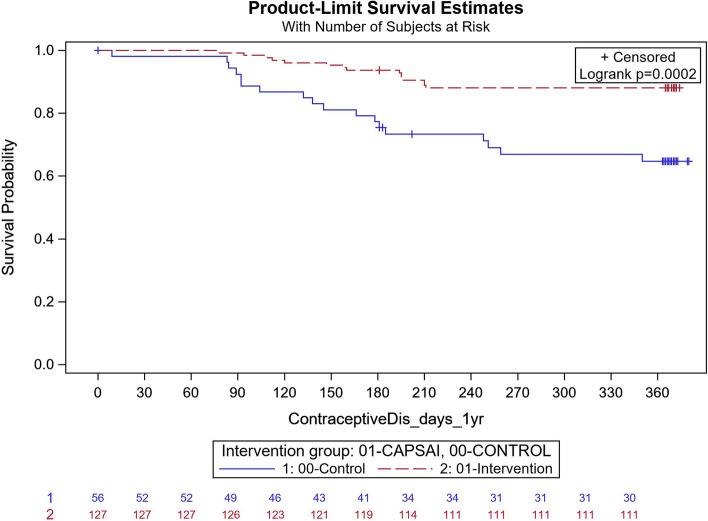
Ghana: contraceptive method discontinuation by 12 months—District level. CaPSAI group: 15 events / 127 at risk and KM survival probability 88.1 (81.1,92.7). Control: 18 events / 56 at risk and KM survival probability 64.7 (49.9,76.2). Frailty model unadjusted HRR CaPSAI vs Control 0.29 (0.14, 0.57), *p*-value = 0.0004

**Fig. 6:**
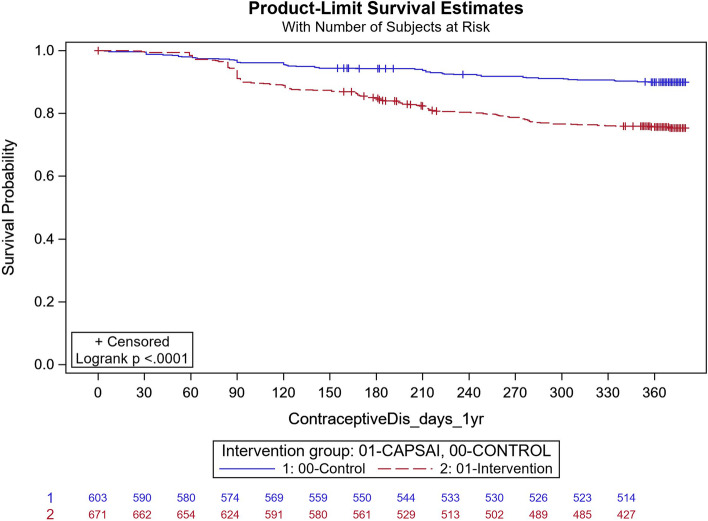
Ghana: contraceptive method discontinuation by 12 months: Health centre. CaPSAI group: 160 events / 671 at risk and KM survival probability 75.3 (71.8,78.5). Control: 59 events / 603 at risk and KM survival probability 89.9 (87.2,92.1). Frailty model unadjusted HRR CaPSAI vs Control 2.39 (1.25, 6.88), *p*-value = 0.0087

In Ghana, in the intervention group, 183 out of 863 participants discontinued their method and, in the control group, 118 out of 822 participants discontinued their method (Fig. 4). Using the frailty model, there was no significant difference between discontinuation in the intervention and control groups (unadjusted HRR = 1.18; 95%CI 0.59,2.37; *p* = 0.64). However, there were significant differences when stratified by type of facility. At district level facilities, 15 out of 127 participants in the intervention group and 18 out of 56 in the control group discontinued their method (Unadjusted HRR = 0.29; 95%CI 0.15, 0.57; *p*-value of 0.0004) (Fig. 5). At the health centre level, 160 of 671 participants in the intervention group and 59 of 603 in the control group discontinued contraceptive method with an unadjusted HRR = 2.42 (95%CI 1.25, 4.69); *p*-value of 0.0087) indicating higher discontinuation in the intervention group (Fig. 6). These results were did not change after adjusting for important confounders (Table 8), there was a 70% reduction in the rate of contraceptive method discontinuation among users at the district hospital level, among participants who were in the intervention group compared to the control group (adjusted HRR = 0.30; 95% CI 0.06,1.37). However, the reverse was the case, for the users at the health centre, where a higher rate of method contraceptive discontinuation was observed in those in the intervention group, relative to those in the control group, with the rate in the intervention group that was almost twice that of the control. (adjusted HRR = 1.98; (95%CI 1.01,3.91).

**Table 8:** Multivariable frailty model Ghana (Outcome = Contraceptive method discontinuation), including group facility level interaction

Factor	Multivariable HRR (95%CI)	MLEChisq *p*-value (btw levels comparison)	Adjusted Type 3 test Chi-sq *p*-value
**Health Facility Service Provider level = **District Hospital			
**Intervention Group**			
CaPSAI	0.30 (0.06, 1.37)		
Control	1.00		
**Health Facility Service Provider level = **Health Centre/Clinic			
**Intervention Group**			
CaPSAI	1.98 (1.01, 3.91)		
Control	1.00		
**Health Facility Service Provider level = **Health Post (Community-based Health Planning Services)/ Maternal/Child Health Clinic/ Other (Home Appointment, Service or visits/Mobile Health Service			
**Intervention Group**			
CaPSAI	0.54 (0.18, 1.59)		
Control	1.00		
**Marital status**			0.063
Married	1.00		
Not married	1.29 (1.02, 1.63)	0.036	
Widowed/Separated/Divorced	0.78 (0.38, 1.60)	0.54	
Group*Health Facility level interaction			0.0026

**Fig. 7:**
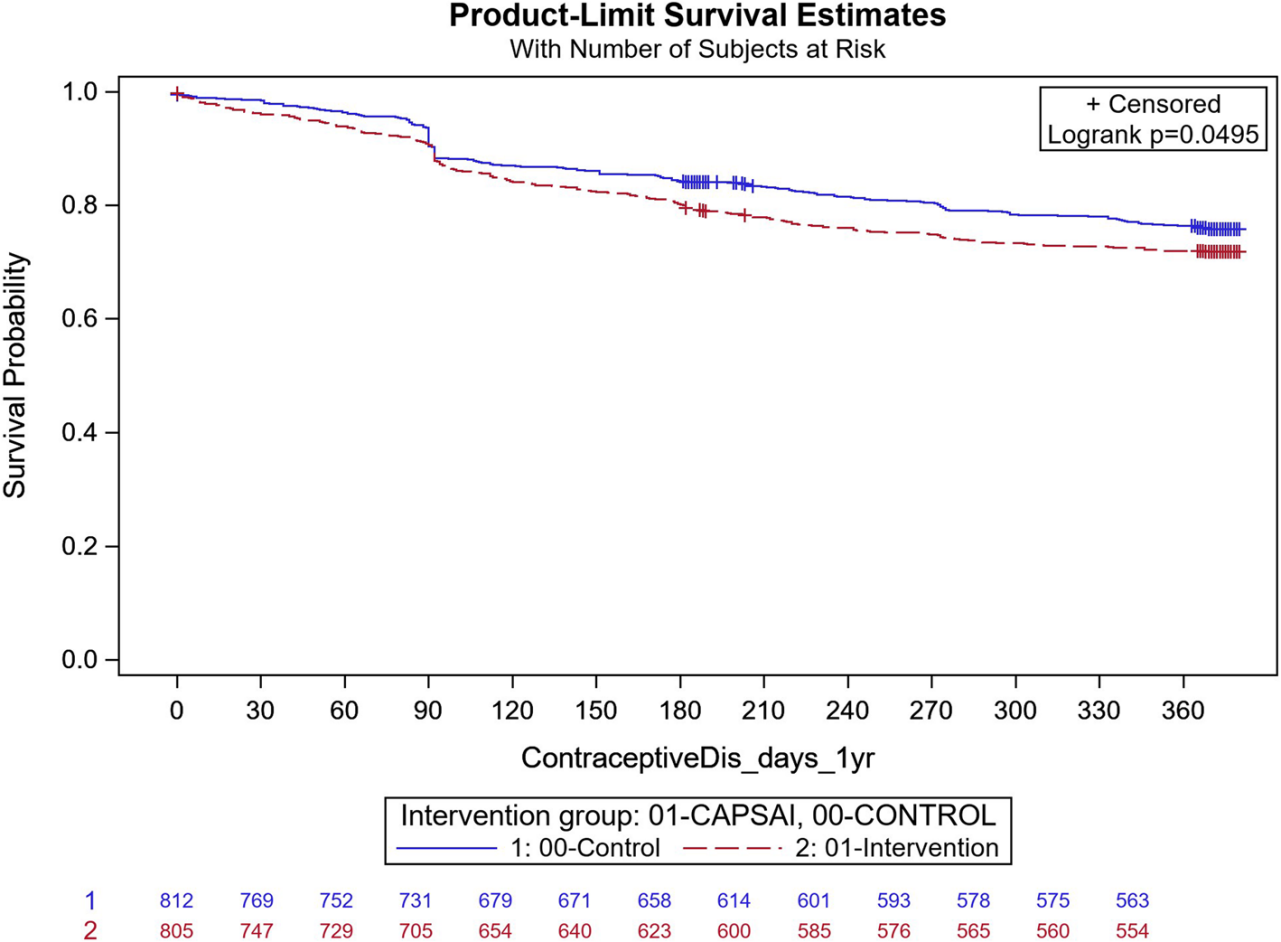
Tanzania: contraceptive method discontinuation by 12 months. CaPSAI group: 184 events / 812 at risk and KM survival probability 71.8 (68.5,74.8). Control: 218 events / 805 at risk and KM survival probability 75.8 (72.6,78.7). Frailty model unadjusted HRR CaPSAI vs Control 1.34 (0.85, 2.12), *p*-value = 0.21

In Tanzania, 184 out of 812 participants discontinued their method in the intervention group,and in the control group, 218 out of 805 discontinued their method. Using the unadjusted Frailty model, there was no significant difference contraceptive method discontinuation in the intervention relative to the control group, (unadjusted HRR 1.34 95% CI 0.85, 2.12; *p* = 0.21) (Fig. 7). The results did not change after multivariable adjustment, in the frailty model with no significant difference in method discontinuation rate in the intervention compared to the control group (adjusted HRR 1.35 95% CI 0.85, 2.12; *p* = 0.21) (Table 9).

**Table 9:** Multivariable frailty model Tanzania (Outcome = Contraceptive method discontinuation)

Factor	Multivariable HRR (95%CI)	MLEChisq *p*-value (btw levels comparison)	Adjusted Type 3 test Chi-sq *p*-value
**Intervention Group**			0.036
CaPSAI	1.35 (0.85, 2.15)	0.21	
Control	1.00		
**Nulligravida**			0.14
Yes	1.76 (0.83, 3.76)	0.14	
No	1.00		

### Method switching

**Fig. 8:**
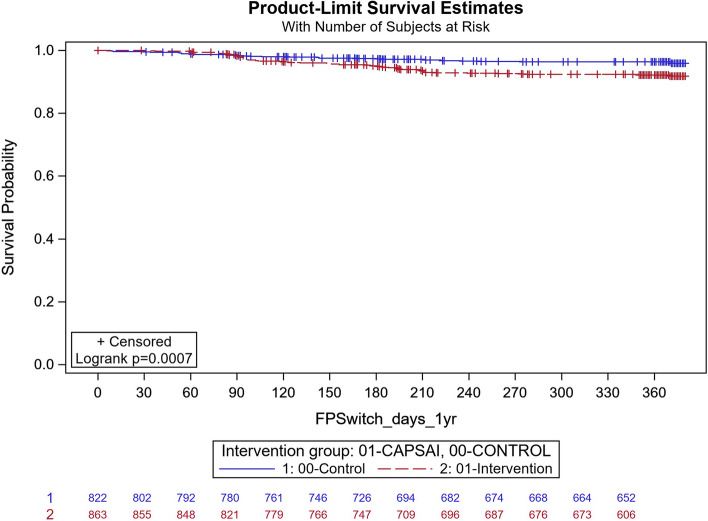
Ghana: contraceptive method switching by 12 months. CaPSAI group: 63 events / 863 at risk and KM survival probability 91.8 (89.6,93.6). Control: 29 events / 822 at risk and KM survival probability 95.9 (94.1,97.2). Frailty model unadjusted HRR CaPSAI vs Control 1.59 (0.80, 3.14), *p*-value = 0.18

**Fig. 9:**
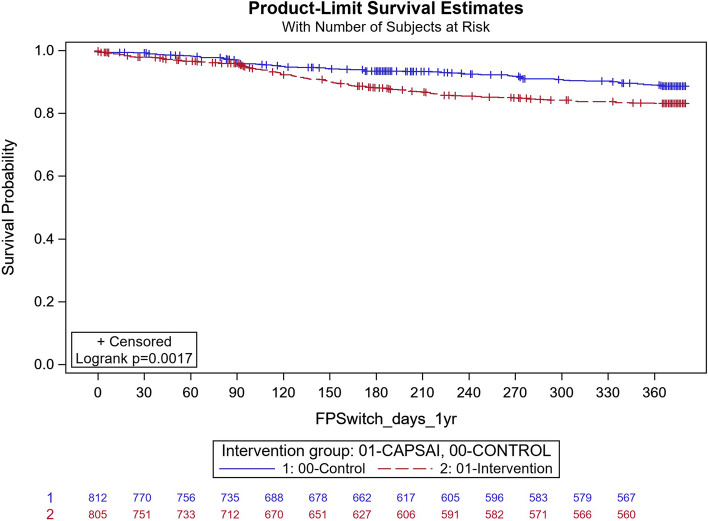
Tanzania: contraceptive method switch by 12 months. CaPSAI group: 80 events / 812 at risk and KM survival probability 83.2 (80.2,85.7). Control: 122 events / 805 at risk and KM survival probability 88.7 (86.1,90.8). Frailty model unadjusted HRR CaPSAI vs Control 1.66 (0.93, 2.96), *p*-value = 0.085

Contraceptive method switching is the proportion of women changing their method within one year of initiation expressed in Fig. 8 and Fig. 9 as time-survival estimates of the intervention against the control group.

In Ghana, in the intervention group, 63 out of 863 participants and 29 out of 822 in the control group switched (Fig. 8). Before adjustment of confounders, the frailty model estimated a 59% increased rate in switching between methods in the intervention group relative to the control group, which was, however, not significant (Unadjusted HRR = 1.59; 95%CI 0.80, 3.14; *p* = 0.18). After multivariate adjustment, the multivariable frailty model showed that the adjusted HRR = 1.55 (95% CI: 0.79, 3.06; *p* = 0.20) (Table 10).

**Table 10:** Multivariable frailty model Ghana (Outcome = method switching)

Factor	Multivariable HRR (95%CI)	MLEChisq *p*-value (btw levels comparison)
**Intervention Group**		
CapSAI	1.55 (0.79, 3.06)	0.20
Control	1.00	
**Years of Education**		
No formal schooling	0.64 (0.24, 1.71)	0.38
Primary Ed {1–6 y (Gh)/ 1–7 y (Tz)}	0.86 (0.39, 1.91)	0.71
Junior/Ordinary Secondary Ed {7–9 y (Gh)/ 8–11 y (Tz)}	1.03 (0.51, 2.11)	0.93
Senior/Advanced Secondary Ed {10–13 y (Gh)/ 12–13 y (Tz)}	0.48 (0.22, 1.07)	0.07
Tertiary Ed {> 13y}	1.00	
**Number of living children**		
0 (none)	1.61 (0.97, 2.67)	0.07
1 or more	1.00	

In Tanzania, in the intervention group, 80 out of 812 participants switched, and in the control group, 122 out of 805 participants switched. Using the univariate frailty model, there was 66% increased switching rate in the intervention group relative to the control, which was however non-significant at 5% level (HRR = 1.66; 95% CI: 0.79, 3.06; *p* = 0.20 *p* = 0.085) (Fig. 9). After adjusting for important confounders in a multivariable frailty model the adjusted HRR = 1.71 (95%CI: 0.96, 3.04; *p* = 0.07) in favor of increased rate in the control group (Table 11).

**Table 11:** Multivariable Frailty Model Tanzania (Outcome = Method switching)

Factor	Multivariable HRR (95%CI)	MLEChisq *p*-value (btw levels comparison)
**Intervention Group**		
CaPSAI	1.71 (0.96, 3.04)	0.07
Control	1.00	
**Age**		
< = 20	1.00	
< = 25	1.61 (1.02, 2.54)	0.043
< = 35	1.47 (0.91, 2.38)	0.11
> 35	0.96 (0.46, 2.01)	0.92
**Occupation**		
Informal sector: Trader/Hawker/Vendor (Informal Business)	1.00	
Formal sector: Professional/Army, Police,Security/Owner Formal Business	0.77 (0.42, 1.43)	0.40
Housewife/Subsistence farmer/Commercial farmer/Labourer/Domestic worker	0.66 (0.45, 0.97)	0.04
Currently not working(its014 = 1) or not working at all (ITS013 = 4)	0.70 (0.49, 0.99)	0.70
**Number of children**		
< 4	0.64 (0.42, 0.98)	0.039
4 +	1.00	

### Contraceptive discontinuation

**Fig. 10:**
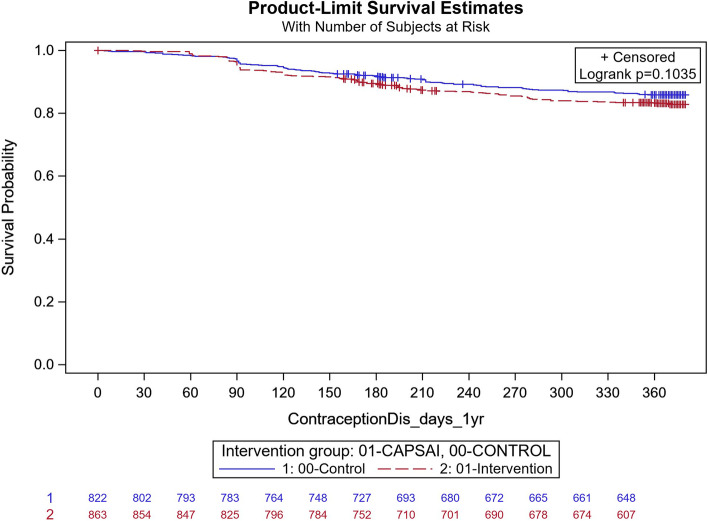
Ghana: contraceptive discontinuation by 12 months. CaPSAI group: 142 events / 863 at risk and KM survival probability 83.4 (80.7,85.8). Control: 111 events / 822 at risk and KM survival probability 85.9 (83.3,88.1). Frailty model unadjusted HRR CaPSAI vs Control 0.95 (0.43, 2.12), *p-value* = 0.91

Contraceptive discontinuation was measured as the proportion of women in the cohort discontinuing contraceptives in the first year of use and expressed in Fig. 10 to Fig. 13 as a time-survival estimate of the intervention against the control group.

**Fig. 11:**
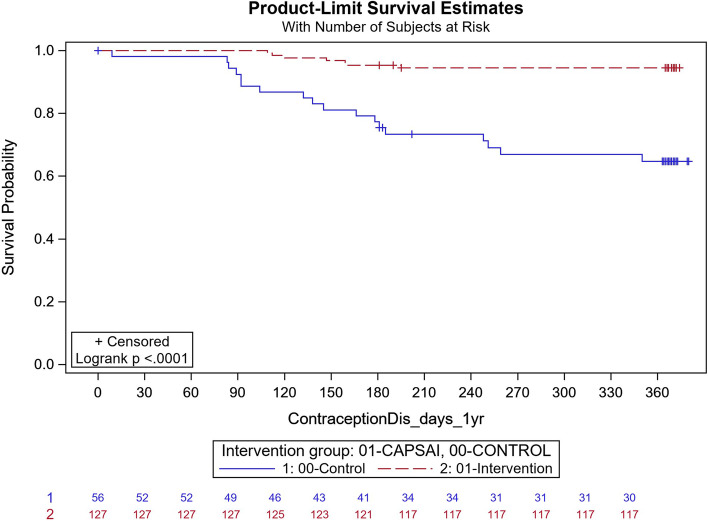
Ghana: contraceptive discontinuation by 12 months – District Hospital. CaPSAI group: 7 events / 127 at risk and KM survival probability 94.5 (88.8,97.3). Control: 18 events / 56 at risk and KM survival probability 64.7 (49.9,76.2). Frailty model unadjusted HRR CaPSAI vs Control 0.13 (0.06, 0.32), *p*-value = 0.0004

**Fig. 12:**
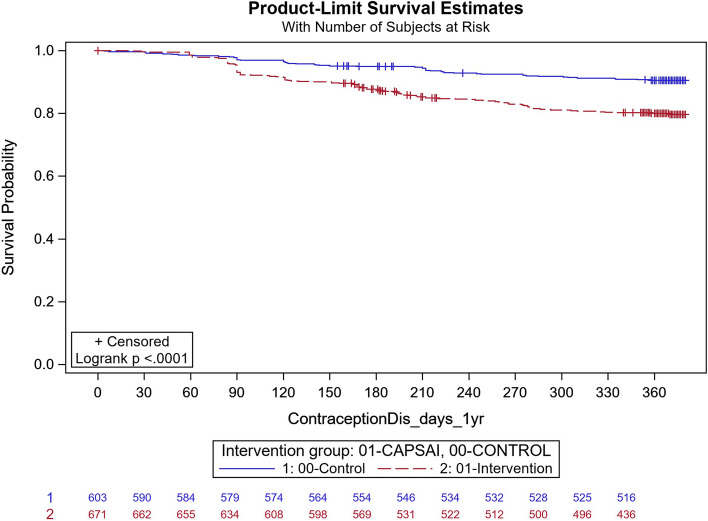
Ghana: contraceptive discontinuation by 12 months – Health Centre. CaPSAI group: 130 events / 671 at risk and KM survival probability 79.6 (76.2,82.6). Control: 55 events / 603 at risk and KM survival probability 90.6 (87.9,92.7). Frailty model unadjusted HRR CaPSAI vs Control 2.07 (0.92, 4.65), *p*-value = 0.078

In Ghana, in the intervention group, 143 out of 863 participants discontinued contraception and, in the control group, 11 out of 822 participants discontinued their method. Using the Frailty model, there was no significant difference between discontinuation in the intervention and control groups (unadjusted HRR = 0.95; 95%CI: 0.43, 2.12; *p* = 0.91) (Fig. 10). However, there were significant differences in contraceptive discontinuation when stratified by type of facility. At the district level, 7 out of 127 participants in the intervention group discontinued their contraception, and 18 out of 56 participants in the control group. At this level, there was a 87% reduction in contraceptive discontinuation in the intervention relative to the control group (unadjusted HRR = 0.13; 95%CI 0.06, 0.32; *p*-value = 0.0004) (Fig. 11). At the health centre level, 130 out of 571 participants in the intervention group discontinued their method, and 55 out of 603 participants in the control group, with the intervention group having twice the rate than that of the control, in discontinuing the contraception method (unadjusted HRR = 2.08; 95%CI:0.92, 4.65; *p*-value = 0.0078) (Fig. 12).

Following adjusting for important confounders (Table 12), there was an 86% reduction in the rate of contraceptive discontinuation among users at the district hospital level, in participants who were in the intervention group relative to the users in the control group (adjusted HRR = 0.14; 95% CI 0.02,0.95). However, the opposite was the case, for the users at the health centre, there was a reported close to the three-quarter increase in the contraceptive discontinuation rate in the intervention relative to the control group, which however did not reach the margin of statistical significance at 5% level (adjusted HRR = 1.72; (95%CI 0.76,3.88).

**Table 12:** Multivariable frailty model Ghana (Outcome = Contraceptive Discontinuation)

Factor	Multivariable HRR (95%CI)
**Health Facility Service Provider level = **District Hospital	
**Intervention Group**	
CaPSAI	0.14 (0.02, 0.95)
Control	1.00
**Health Facility Service Provider level = **Health Centre/Clinic	
**Intervention Group**	
CaPSAI	1.72 (0.76, 3.88)
Control	1.00
**Health Facility Service Provider level = **Health Post (Community-based Health Planning Services)/ Maternal/Child Health Clinic/ Other (Home Appointment, Service or visits/Mobile Health Service	
**Intervention Group**	
CaPSAI	0.33 (0.09, 1.27)
Control	1.00
**Nulligravida**	
Yes	1.36 (0.94, 1.94)
No	1.00
Group*Health Facility level interaction	

**Fig. 13:**
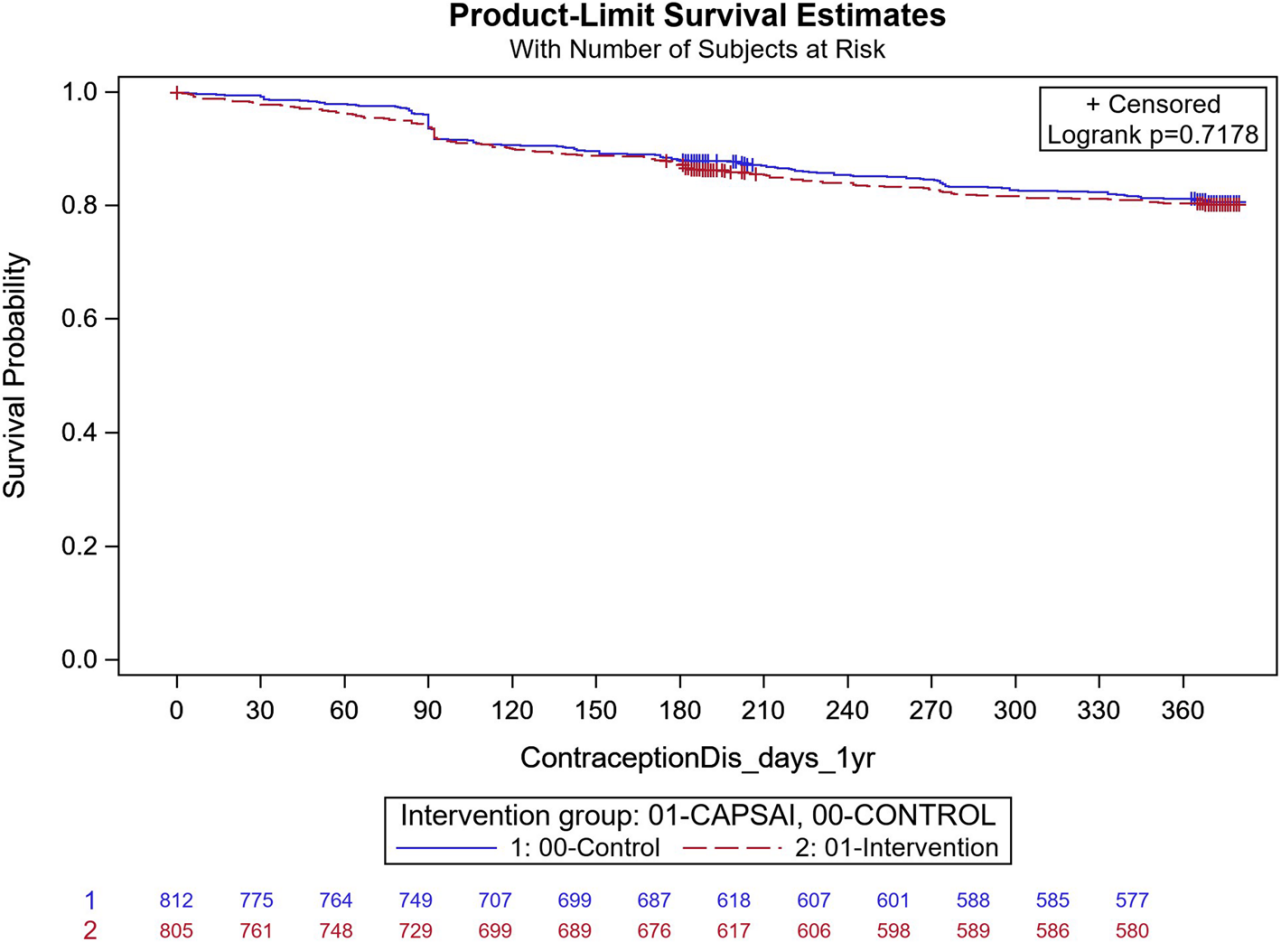
Tanzania: contraceptive discontinuation by 12 months. CaPSAI group: 145 events / 812 at risk and KM survival probability 80.2 (77.2,82.9). Control: 150 events / 805 at risk and KM survival probability 80.6 (77.6,83.3). Frailty model unadjusted HRR CaPSAI vs Control 1.13 (0.66, 1.91), *p*-value = 0.66

In Tanzania, 145 out of 812 participants discontinued their method in the intervention group, and in the control group, 150 out of 805 participants discontinued their method. Using the Frailty model, there was no significant difference between discontinuation in the intervention and control groups (unadjusted HRR = 1.13; 95% CI: 0.66, 1.91; *p* = 0.66) (Fig. 13). After adjusting for important confounders in a multivariable frailty model, the adjusted HRR = 1.04 (95%CI: 0.64, 1.70) was not statistically significant difference between the two groups (Table 13).

**Table 13:** Multivariable frailty model Tanzania (Outcome = Contraceptive discontinuation)

Factor	Multivariable HRR (95%CI)
**Intervention Group**	
CaPSAI	1.04 (0.64, 1.70)
Control	1.00
**Type of Health facility**	
Health centre/clinic	0.72 (0.45, 1.14)
Dispensary	1.00
**Education level (based on years of education)**	
Primary or lower	1.27 (0.98, 1.66)
Secondary or higher	1.00
**Gravida**	
0	3.34 (1.49, 7.51)
1	1.09 (0.77, 1.52)
2	0.97 (0.69, 1.36)
3	1.07 (0.75, 1.53)
4 +	1.00

### Reasons for discontinuation in methods at follow up interview

In Ghana, the most important reasons reported for stopping a method were fear of side-effects (34.1%), health concerns (13.6%) and wanting to become pregnant (13.6%) in the control group and fear of side-effects (25.8%), wanting a more effective method (17.7%) and infrequent sex (16.7%) in the intervention group (Table 14). In Tanzania, the most important reasons reported for stopping a method were fear of side-effects (24.6%), wanting a more effective method (18%), and method not available (18%) in the control group compared to wanting a more effective method (27.6%), fear of side-effects (17.2%) and health concerns (17.7%) in the intervention group (Table 14).

**Table 14:** Main reason for discontinuation in Ghana and Tanzania

Ghana:			
What was the most important reason you stopped using the method	Control *n* = respondents (%)	Intervention *n* = respondents (%)	Total respondents
Fear of side effects	15 (34.1)	16 (25.8)	31
Infrequent sex/husband away	4 (9.1)	10 (16.1)	14
Wanted more effective method	1 (2.2)	11 (17.7)	12
Health concerns	6 (13.6)	5 (8.1)	11
Wanted to become pregnant	6 (13.6)	4 (6.5)	10
Other	12 (27.3)	16 (25.8)	28
Total	44	62	106
**Tanzania:**			
What was the most important reason you stopped using the method	Control *n* = respondents (%)	Intervention *n* = respondents (%)	Total respondents
Wanted more effective method	11 (18.0)	16 (27.6)	27
Fear of side effects	15 (24.6)	10 (17.2)	25
Health concerns	6 (9.8)	10 (17.2)	16
Wanted to become pregnant	7 (11.5)	5 (8.6)	12
Method not available	11 (18.0)	1 (1.7)	12
Other	11 (18.0)	16 (27.6)	27
Total	50	58	119

### Satisfaction

In both countries, questions eliciting satisfaction with the provider care and facility care yielded a very high positive response with the intake. They could not be analysed to see differences over time (Table 15 and Table 16). A high percentage (close to 100%) of women said that they would return to the same provider during the intake interview in both control and intervention groups. The same was found when women were asked if they would refer a friend or a relative to the facility or provider.

**Table 15:** Questions eliciting satisfaction with provider and facility (Ghana)

	Control		Intervention	
Questions asked	Intake interview	Follow-up interview	Intake interview	Follow-up interview
**Would you return to the same provider?**	Yes = 502 (98.8%) No/unsure = 6 (1.2%) (*n* = 508)	Yes = 493 (97.7%) No/unsure = 15 (2.95%) (*n* = 508)	Yes = 584 (99.7%) No/unsure = 2 (0.4%) (*n* = 584)	Yes = 574 (97.1%) No/unsure = 12 (2.05%) (*n* = 584)
**Would you refer your relative or friend to this provider/facility?**	Yes = 496 (97.6%) No/unsure = 12 (2.4%) (*n* = 508)	Yes = 486 (95.7%) No/unsure = 22 (4.3%) (*n* = 508)	Yes = 537 (91.5%) No/unsure = 47 (8.1%) (*n* = 584)	Yes = 547 (93.7%) No/unsure = 37 (6.34%) (*n* = 584)

**Table 16:** Questions eliciting satisfaction with provider and facility (Tanzania)

	Control		Intervention	
Questions asked	Intake interview	Follow-up interview	Intake interview	Follow-up interview
Would you return to the same provider?	Yes = 244 (99.2%) No/unsure = 2 (0.8%) (*n* = 246)	Yes = 244 (99.2%) No/unsure = 2 (0.8%) (*n* = 246)	Yes = 317 (97.8%) No/unsure = 7 (2.1%) (*n* = 324)	Yes = 321 (99.1%) No/unsure = 3 (0.9%) (*n* = 324)
Would you refer your relative or friend to this provider/facility?	Yes = 244 (99.6%) No/unsure = 1 (0.4%) (*n* = 245)	Yes = 244 (99.6%) No/ unsure = 1 (0.4%) (*n* = 245)	Yes = 313 (96.6%) No/unsure = 11 (3.4%) (*n* = 324)	Yes = 320 (93.7%) No/unsure = 4 (1.2%) (*n* = 324)

## Discussion

The study's overall aim is to demonstrate how a social accountability process in family planning and contraceptive -programs and -services influence Quality of Care and client satisfaction and whether this leads to increased contraceptive uptake and use. We report here on the relationship between social accountability and the use of modern contraceptives, i.e., contraceptive method discontinuation, contraceptive method switching, and contraceptive discontinuation. To our knowledge, this is the first study reporting on contraceptive use after a social accountability intervention. In this cohort study over a one-year duration, we did not find a statistically significant difference in Ghana and Tanzania in overall method discontinuation, switching, and contraceptive discontinuation after exposure to a social accountability intervention.

### Differences in Ghana when stratified according to the level of facility and the health system structure

However, in Ghana but not in Tanzania, when stratified by the type of facility (district level vs. health centre), there were significantly less method and contraceptive discontinuation in the district level facility and significantly more method and contraceptive discontinuation in the health centres in the intervention group. These findings validate our ToC and previous study findings that social accountability is context-driven process, and understanding the different factors, including the health care system structure, is key to evaluating their effect. Health systems are made up of complex networks of interconnected actors that influence service delivery at varying degrees [28]. Policy decisions and institutional organizations and procedures may affect how interventions change contraceptive use. Meanwhile, the interface between infrastructure, clients, and providers determines the quality of services. Several health system characteristics have been identified as enabling social accountability. Decentralization of governance and service delivery structures are essential as it puts priority setting closer to the community [28]. When the decentralization processes are not fully realized, local government systems, cannot meaningfully engage with clients and community members. They may not have the capacity or the resources to respond to the demands resulting from the social accountability process [39, 40].

In the case of Ghana, significant differences in the unexpected direction" were demonstrated when stratified by facility type. This may be explained through the health system perspective, where less discontinuation is expected at the health centre level as communities are assumed to have closer interaction with the health centre providers, both in terms of services but also through family planning and reproductive health activities and mechanisms. Meanwhile, it is also expected that district hospitals will have a higher cadre of health personnel who can offer more elaborate FP methods such as IUD compared to lower cadre of health personnel who staff health centres and CHPS compounds at a lower level. We found less discontinuation in district hospitals which are likely to offer a variety of methods and higher cadre of personnel [41–43].

The state may also not be the only provider of contraceptive services. In many settings, commercial service providers and non-profit organizations also provide family planning [44]. These non-state actors may also influence family planning decision-making and use. For CaPSAI, context mapping in-depth interviews were conducted at the district level in both countries to capture these family planning services done by non-state actors but will be reported elsewhere.

### Method choice and continuation

These interventions are context-specific, as shown by the methods of choice at the initiation of enrollment [45, 46]. In this study, in Ghana, women overwhelmingly chose injectables followed by implants, and in Tanzania, it was implants followed by injectables. These are in line with existing method prevalence data in both countries [33]. Continuation rates are different between the two methods, with implants having a higher continuation rate [47]. Despite the differences in method mix, overall method discontinuation was not affected in both countries suggesting no differential impact of SA intervention by method used.

There is no clear reason why this was the case. Possible explanations could be explored, include that the intervention was not long enough sustained and the intermediate effects, such as behavioural changes, including myths and structural changes have not taken hold or that there was not enough time before the intervention was evaluated to observe these changes. Another explanation may be that the pathway of the Theory of Change did not lead to the expected changes. Lastly commodity availability may have influenced the results. The drivers are being explored further in the process evaluation which will be published separately [25].

### Informed choice: differences in the two groups

On provider behavior to enable women to make an informed choice the study found variability by behavior and country. In Ghana, after the intervention, the provider was more likely to give information on method options but gave better counseling on side effects in the control group. There were no significant differences on whether health providers gave women an opportunity to ask questions and respond to questions.

In Tanzania, the findings from the informed choice questions were significantly better in the control group except for one question (Table 5). These findings could be due to an impact of the SA intervention on provider behavior or women being more demanding or less satisfied with the status quo [48]. There can also be differences because of contextual factors such as outreach and training provided by NGOs to facility staff. This will be further analysed through the context mapping and intermediate outcomes and will be reported elsewhere.

### Satisfaction of family planning care and services

In both countries and in both groups, there were overall high rates of "very satisfied" and "satisfied" responses before and after the intervention. This high level of satisfaction may have limited the interventions’ impact on contraceptive use by improving satisfaction. The assumption in the ToC that there was low satisfaction whose improvement would be a pathway through which contraceptive use is affected did not hold in these particular contexts.

### Importance of intermediate outcomes and process evaluation

The reasons why women discontinued remained the same in intervention and control, suggesting a limited impact of the intervention on them. In Ghana, the main reasons for discontinuation in the control group and intervention group were fear of method side-effects. In the intervention group, other main reasons were wanting a more effective method and infrequent sex. In Tanzania, the most important reasons reported for stopping a method were fear of side-effects in the control group and wanting a more effective method followed by fear of side-effects in the intervention group. This is in keeping with other studies showing that side effects are major reasons for the discontinuation of methods [49]. This is also reflected in the wider literature on family planning and contraceptive discontinuation [49, 50]. In a review, reasons for discontinuing included the reduced need for family planning, which may include changes in fertility status and fertility intentions, infrequent sex [7, 50]. Among discontinuers who still need, reasons for stopping their contraception include becoming pregnant despite being on a method, health concerns, those who switch to a more effective method, lack of access to their method, and husband or partner opposition [50]. In Ghana, a national-level survey revealed that the top three reasons why women discontinue use of contraceptives were wanting to become pregnant (27%), side effects/health concerns (21%), and becoming pregnant while using (20%) [31]. In Tanzania, the most common reason for discontinuing a method in less than 12 months is the desire to become pregnant (38%), followed by method-related side effects or health concerns (26%) [33]. Other common reasons are infrequent sex, inconvenient to use, health concerns, desire to become pregnant, difficulty in getting pregnant, menopause, marital dissolution, and method failure were frequently reported by other studies [51, 52].

To understand the link between social accountability and contraceptive use as reported in this paper, there is need to: evaluate how women make decisions, their self-efficacy, knowledge, attitude and practices, their interaction with service providers, the accessibility and availability of methods, norms, and gender dynamics in specific settings, and how these are affected by social accountability processes. CaPSAI captured the impact of social accountability on some of these issues and these findings will be reported elsewhere.^1^ Specifically, a cross-sectional survey with validated psychometric scales explored the links between social accountability interventions and service users perceptions of empowerment, efficacy, and engagement with the health care providers [25]. Within and across the two countries, the results were mixed whereby two domains registered positive changes, while five domains registered negative changes over time and four reversed directions during the study period. Moreover, a process evaluation that included case studies of change aimed to capture the changes related to the intervention and collected data to determine what factors were present and key for a change to take hold^1^ [24].

### Limitations

We selected facilities/districts that were comparable for basic characteristics (level and number of users), but there may be differences between the groups that we did not account for. There are clear differences between the countries, but the basic demographics are similar between the groups within a country. In general, there were similar demographic characteristics between the control and the intervention groups in both countries. However, further analysis of the contextual factors, i.e. ongoing facility-led outreach and NGO activities, are needed to examine the comparability of the groups and will be reported elsewhere^1^.

Even though the follow up rate is very impressive for a study conducted in these countries, there is also a major difference in follow-up rates in the same facilities between the countries. It is more important to note that many women, especially in Tanzania, did not use the same facility where they were recruited during the intake interview. Although the women responded that they were not anticipating to move outside the intervention area/district during the period of study during the screening, this was the leading reason for attending a different facility at follow up.

Knowledge and exposure to the intervention were low in the intervention groups of both Ghana and Tanzania at the intake as well as the follow-up interview. According to the ToC, this should not have affected the study as the intervention had its effect at the facility level resulting in changes that would enhance the use of contraceptive methods. There is also a risk of under-estimation bias if, despite intervention coverage in the intervention facility/catchment area, some women choose to go to one facility over another facility in the same catchment area.

Processes that require changes in behavior, including social accountability interventions, take time to be effective and behaviour change maintenance depends on motives, self-regulation, resources (psychological and physical), habits, environmental and social influences [53, 54]. Both these timing of the measurement and maintenance of the behavioural change may have played a role in the outcomes measured here.

Qualitative data at the community level to understand social and gender dynamics in the context of social accountability was not purposefully collected due to budgetary constraints for a more intensive process evaluation. Firstly, the CaPSAI study process evaluation focused on collecting data at the district level in both intervention and control groups to understand family planning initiatives and other community participation programs as part of the context mapping^1^. Secondly, non-participant observation of key intervention activities and in-depth interviews were conducted in four of the eight interventions sites in each country with intervention participants to trace the implementation and gain a fuller description of the social accountability process and how the outcomes were produced^1^. Finally, case studies were conducted to retrospectively explore reported changes resulting from the intervention^1^.

## Conclusion

We did not demonstrate a statistically significant impact of a six-month CaPSAI intervention on contraceptives use among new users in Tanzania and Ghana. However, since social accountability have important impacts beyond contraceptive use it is important consider results of the intermediate outcomes, cases of change, and process evaluation to fully understand the impact of this intervention. Studies to understand potential links between SA and service utilization should include counterfactual analysis and be supported by structured process evaluation.

Social accountability is complex and may result in complex actions which may not be straightforward to explain. A social accountability intervention could lead to women who have been exposed to a social accountability-related activity resulting in more demanding or less satisfied with the status quo. Social accountability may not consistently decrease discontinuation and its impact are complicated by other health system and contextual factors, including the responsiveness of the health care system such as the level of service, including setting.

## Data Availability

The de-identified dataset used and/or analysed during the current study can be requested from the Primary Sponsor or Principal Investigators, and data will be shared contingent on approval by the internal review and approval by local internal ethics review board.

## Footnotes

1 All published manuscripts related to the Community and Provider driven Social Accountability Intervention (CaPSAI) Project are and will be uploaded on the Australian New Zealand Clinical Trials Registry (ACTRN12619000378123).
